# Harnessing the natural inhibitory domain to control TNFα Converting Enzyme (TACE) activity *in vivo*

**DOI:** 10.1038/srep35598

**Published:** 2016-12-16

**Authors:** Eitan Wong, Tal Cohen, Erez Romi, Maxim Levin, Yoav Peleg, Uri Arad, Avraham Yaron, Marcos E. Milla, Irit Sagi

**Affiliations:** 1 Molecular Pharmacology and Chemistry Program, Memorial Sloan Kettering Cancer Center, New York, NY, USA; 2 Department of Biological Regulation, Weizmann Institute of Science, Rehovot, Israel; 3 Department of Biological Chemistry, Weizmann Institute of Science, Rehovot, Israel; 4 The Israel Structural Proteomics Center, Weizmann Institute of Science, Rehovot, Israel; 5 Department of Rheumatology, Tel Aviv Sourasky Medical Center and the Sackler Faculty of Medicine, Tel Aviv University, Israel; 6 Janssen Research & Development, San Diego, CA, USA.

## Abstract

Dysregulated activity of A Disintegrin And Metalloproteinase 17 (ADAM17)/TNFα Converting Enzyme (TACE) is associated with inflammatory disorders and cancer progression by releasing regulatory membrane-tethered proteins like TNFα, IL6R and EGFR ligands. Although specific inhibition of TACE is thought to be a viable strategy for inflammatory disorders and for malignancies treatment, the generation of effective inhibitors *in vivo* has been proven to be challenging. Here we report on the development of a protein inhibitor that leverages the endogenous modulator of TACE. We have generated a stable form of the auto-inhibitory TACE prodomain (TPD), which specifically inhibits *in vitro* and cell-surface TACE, but not the related ADAM10, and effectively modulated TNFα secretion in cells. TPD significantly attenuated TACE-mediated disease models of sepsis, rheumatoid arthritis (RA) and inflammatory bowel disease (IBD), and reduced TNFα in synovial fluids from RA patients. Our results demonstrate that intervening with endogenous ADAM sheddase modulatory mechanisms holds potential as a general strategy for the design of ADAM inhibitors.

Members of the A Disintegrin And Metalloproteinase family (ADAMs) are multidomain type I transmembrane proteinases responsible for ectodomain proteolytic processing of membrane-tethered proteins (shedding). These proteins play important roles in many biological processes such as embryonic development^1^,^2^, inflammatory responses^3^,^4^, pathogenesis of cancer^5^, and Alzheimer’s disease^6^,^7^,^8^. Within this family, ADAM17 occupies a central place in pathophysiology. Originally identified as the major enzyme for TNFα release (TNFα Converting Enzyme or TACE)^9^,^10^, later studies revealed that this zinc metalloproteinase is also responsible for the processing of cell adhesion proteins (e.g. L-selectin and ICAM1), cytokine receptors (e.g. IL6R and TNFR), and ligands of EGF receptors^11^. Transgenic mice expressing an inactive form of TACE exhibit deficits that are strikingly similar to those observed with TGF receptor knockout mice^12^, demonstrating TACEs role in TGFα processing. While TACE is indispensable during development, its active cell surface form is mainly found in adulthood during inflammation and cancer^13^,^14^,^15^.

Dysregulation of ectodomain shedding is associated with infection, inflammation, autoimmune and cardiovascular diseases, neurodegeneration and cancer^16^. Various studies have indicated that TACE plays multiple pivotal pro-tumoral roles^11^,^13^,^15^. In particular, it activates many of the ligands that bind to members of the ErbB tyrosine kinase family of receptors, which are involved in the growth of many tumors^17^. For example, EGFR ligands such as amphiregulin, epiregulin, epigen and TGFα are activated by TACE. Moreover, the involvement of TACE in inflammation was demonstrated in case studies of human TACE deletion^18^,^19^. The patients exhibited inflammatory lesions in the skin and intestine and acute hyper-inflammation caused by increased susceptibility to opportunistic infections due to impaired in cytokine secretion. Therefore, inhibition of TACE-mediated shedding activity may be therapeutically beneficial in the treatment of inflammation and cancer^14^,^15^.

TACE is biosynthesized as a zymogen and activated upon proteolytic release of its auto-inhibitory prodomain^20^,^21^. The prodomains, in a similar manner to the tissue inhibitors of metalloproteinases (TIMPs), employ extensive protein-protein surface interaction and cysteine coordination with the catalytic metal ion, effectively blocking protease activity^22^,^23^,^24^,^25^. TACE prodomain behaves as an independent folding unit and acts as an effective inhibitor of TACE *in vitro*^23^. Previous studies were limited due to difficulties in the production of natively folded, functional TACE prodomain. Expression of ADAM prodomains formed insoluble inclusion bodies in *E. coli*, requiring *in vitro* refolding after chemical denaturation. The refolded TACE prodomain exhibited low thermodynamic stability, exemplified by its tendency to aggregate. *In vitro* the refolded TACE prodomain did not effectively inhibit the endogenous cell-associated TACE ectodomain, further questioning its potential as TACE specific inhibitor^23^. Consequently, employment of the isolated TACE prodomain as an exogenous modulator *in vivo* has not been fully explored thus far^26^,^27^.

By performing gene optimization, we have succeeded in producing scale-up amounts of correctly folded, stable and functional human TACE prodomain (TPD). We show that TPD is a potent, highly selective and efficacious *in vivo* modulator of both human and mouse TACE sheddase activity. Thus, harnessing an endogenous inhibitory mechanism for reconstitution of the TACE zymogen *via* exogenous addition of its natively folded prodomain is a potential approach for protein-based inhibitor design. Importantly, the TACE prodomain shares almost no sequence homology to other related ADAMs and extremely poor homology to other MMP prodomains, making TACE prodomain a good candidate as a specific inhibitor of TACE.

## Results

### Regeneration of TACE prodomain (TPD) and *in vitro* inhibition

**Figure 1:**
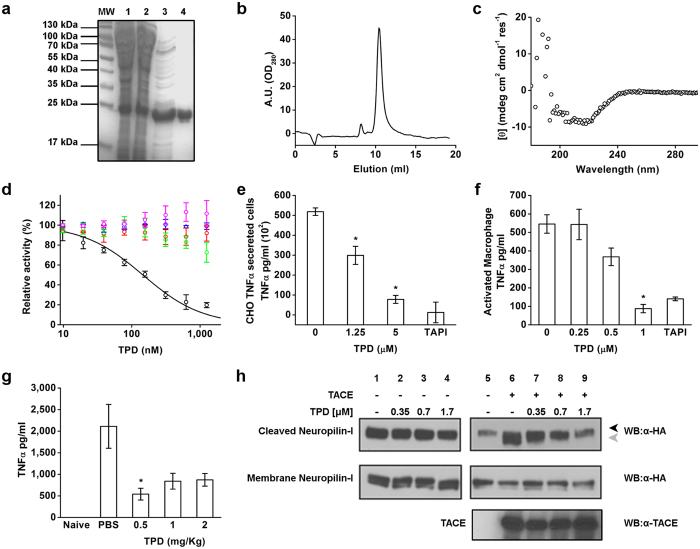
The natively folded TPD inhibition *in vitro* and modulation of TNFα secretion in cell based assay and *in vivo*. (**a**) Representative purification procedure of recombinant TPD: Molecular weight ladder (MW), whole bacteria lysate (1), lysate soluble fraction (2), Ni+ NTA elution (3) and anion exchange first peak elution (4). (**b**) Purified TPD eluted from gel filtration superdex 75 in a sharp single peak. (**c**) Circular dichroism measurement of purified TPD in room temperature. (**d**) Catalytic activity of TACE (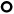), MT1-MMP (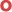), MMP7 (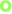), MMP9 (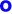) and ADAM10 (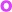) on fluorogenic peptide in presents of TPD. Calculated IC_50_ value of TACE is 145 ± 1.9 nM and up to 2000 nM no significant inhibition was observed for other catalytic activity. (**e**) Secretion of TNFα in CHO stable transfected TNFα cells incubated with TPD treatment. *P < 0.05, n = 4 (Mean ± SD, Student’s *t* test) (**f**) Secretion of TNFα in primary activated macrophages. LPS is used to stimulate the macrophages prior to treatment. TAPI (small molecule inhibitor) in 20 μM concentration is used as positive control in both cell types assays. *P < 0.05, n = 3 (Mean ± SD, Student’s *t* test). (**g**) Serum level of TNFα in naive C57/BL mice and C57/BL mice injected with LPS and treated with control PBS or different concentrations of TPD (0.5, 1, 2 mg/Kg). *P < 0.05, n = 8 (Mean ± SEM, Student’s *t* test). (**h**) Shedding of Neuropilin-1 from HEK293 cells. Transfection of Neuropilin-1 only in the presence of TPD (lane 1–4) shows no effect on the shedding pattern by endogenous ADAM10. Overexpressing both Neuropilin-1 and TACE in the present of TPD (lane 5–9), generates a specific cleavage by TACE which is inhibited by TPD in a concentration-dependent manner. The black arrow indicates the cleavage product by ADAM10 and the grey arrow the cleavage product of TACE.

Engineering a stable TACE prodomain has proved to be difficult, in our hands as well as in others, due to poor solubility^23^,^28^. That procedure required refolding of the prodomain from inclusion bodies under elaborate conditions. To produce a more stable and soluble TACE prodomain, we screened various constructs and expression conditions, and optimized the primary sequence for cytosolic expression in *E. Coli*. From these efforts, we identified a form comprising residues Asp^23^-Arg^214^ which resulted in an expression of soluble, natively folded protein in the cytosolic fraction. A three-step purification procedure including affinity, ion exchange and size exclusion chromatography yielded >95% homogeneous protein (Fig. 1a). The resulted extra band seen in Fig. 1a lane 4 is often a truncated form lacking the His tag. Purified TACE prodomain (TPD) is eluted in size exclusion chromatography in a single sharp peak, demonstrating a mono-disperse protein sample (Fig. 1b). TPD showed substantial secondary structure as shown by monitoring of its circular dichroism (CD) in the far UV region (Fig. 1c). To profile TPD functionally, we performed inhibition studies against TACE and a panel of related matrix metalloprotease (MMP) family members, as well as the close related ADAM10. TPD inhibited the enzymatic activity of recombinant TACE catalytic domain *in vitro* with an IC_50_ = 145 ± 1.9 nM, yet it lacked potency against ADAM10, MMP14 and MMP9, and showed less than 10% inhibition at MMP7 at concentration of up to 2 μM (Fig. 1d).

### TPD modulates excessive TNFα shedding in human and mouse cells as well as in *in vivo* LPS induced septic shock model

To determine whether TPD effects the shedding of TACE substrates, we employed a recombinant CHO M1 cell line stably expressing human WT TACE and TNFα^29^ and LPS-stimulated mouse macrophages. TPD inhibited TNFα release from CHO M1 cells in a dose-dependent fashion (85% inhibition at 5 μM and 40% inhibition at 1.25 μM) (Fig. 1e). Similarly, exogenous treatment with human TPD inhibited LPS induction of TNFα release from primary macrophages in a dose dependent manner (Fig. 1f), with 80% inhibition at 1 μM. This result is not surprising because human TACE prodomain and its mouse homolog are highly similar, sharing 85% amino acid homology. Thus, TPD is a potent modulator of human and mouse endogenous cell-associated TACE related ectodomain shedding. The ability of TPD to modulate TACE activity *in vivo* was tested in an acute LPS induced septic shock model in Balb/c mice, where TACE was shown to play a central role by effecting the release of TNFα^30^. Remarkably, treatment with 0.5 mg/Kg of TPD led to over 80% decrease in TNFα levels in the serum (Fig. 1g). The higher doses of 1 and 2 mg/Kg gave less reduction in TNFα secretion (60%).

### Selective activity of TPD towards TACE and not towards the close related ADAM10 in cells

Within the ADAM family, TACE prodomain is most homologous to ADAM10 prodomain, however sharing sequence homology of only 23%. Nevertheless, isolated ADAM10 prodomain inhibited TACE catalytic domain *in vitro*^25^. To test the specificity of TPD against ADAM10, we applied a cell-based assay using the differential cleavage of the receptor Neuropilin-1^31^,^32^,^33^. Neuropilin-1 is a membrane-bound co-receptor to a receptor tyrosine kinase, shed by both TACE and ADAM10 at two distinct sites^32^. HEK293 cells, constitutively expressing ADAM10, were transiently transfected with C-terminally HA-tagged Neuropilin-1, exhibited a distinct cleavage product in the cell media detected by anti-HA antibodies (Fig. 1h). Importantly, ADAM10 mediated cell-surface shedding activity of Neuropilin-1 was not affected by the addition of TPD (Fig. 1h, lines 1–4). However, when HEK293 cells were co-transfected with Neuropilin-1 and TACE, an additional cleavage product with lower molecular weight was found (Fig. 1h, line 6) a result of TACE distinct shedding activity. In contrast to ADAM10, TACE-mediated Neuropilin-1 receptor shedding was blocked by TPD in a dose dependent manner (Fig. 1h, line3–5) under similar conditions, reinforcing the selective inhibition of TPD towards TACE but not ADAM10.

### Modulation of pro-TNFα maturation *in vivo* by TPD resulted in effective therapy in inflammatory and autoimmune disease models

**Figure 2:**
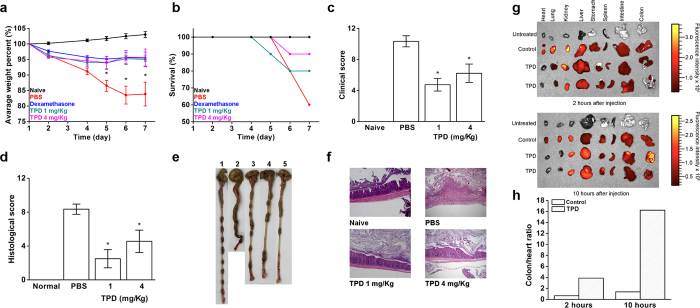
Treatment with TPD protects against TNBS colitis development. Clinical colitis severity was monitored by weight loss (**a**), survival rate (**b**) (dexamethasone overlaps with the TPD 4 mg/kg curve and it is not apparent in the graph), clinical score (**c**), histopathologic analysis performed in hematoxylin/eosin-stained sections of colons (**d**), representative colons feature groups (**e**) (1- naïve, 2- PBS, 3- Dexamethasone, 4- TPD 1 mg/Kg, 5- TPD 4 mg/Kg) and histologic features of representative colonic sections at x10 magnification (**f**) of TNBS induced mice treated with either PBS, Dexamethasone as positive control and TPD 1 or 4 mg/Kg. *P < 0.05, n = 10 (Mean ± SEM, Student’s *t* test). Representative images of dissected organs (**g**) of TNBS treated or healthy mice sacrificed 2 h or 10 h after intravenous injection of fluorescently labeled TPD (1.5 nmol of TACE prodomain-HiLyte Fluor 750) or fluorescent dye only (1.5 nmol of Hilyte Fluor 750). (**h**) Colon-to-heart ratios of the fluorescence for the mice sacrificed 2 and 10 hours after injection.

TNFα has a causative role in the pathogenesis of chronic auto-inflammatory diseases such as rheumatoid arthritis (RA) and inflammatory bowel disease (IBD). To further explore the modulating activity of TPD, we investigated its therapeutic effect in TNBS-induced colitis (IBD model) and collagen induced arthritis (CIA, RA model). Mice subjected to TNBS developed severe illness with anticipated symptoms that included bloody diarrhea and severe weight loss (16%) and upon treatment with 1 mg/Kg and 4 mg/Kg by daily intravenous injections of the TPD, animals exhibited lower weight loss of 4% and 5%, respectively (Fig. 2a). The survival rate was further improved with the injection of TPD (Fig. 2b). The efficacy of TPD’s inhibitory activity was also apparent through an improvement in the clinical and histological scores (Fig. 2c, d). TPD-treated mice displayed a longer colon length, solid feces and fewer lesions (Fig. 2e). Histopathology revealed that TPD treatment prevented massive infiltration of macrophages and immune cells to the colon lumen as seen in the PBS control (Fig. 2f). Moreover, 10 hours after administration, TPD localized in the colon of treated animals, as suggested by fluorescence imaging analysis of fresh tissues using labeled TPD (Fig. 2g). In contrast, the unconjugated fluorescent label control did not accumulate in the colon. The colon to heart ratio of the fluorescent intensity is 1.3 fold compare to 16.2 fold in the TPD (Fig. 2h). This suggests that TPD may specifically localize to inflamed tissue in this model.

**Figure 3:**
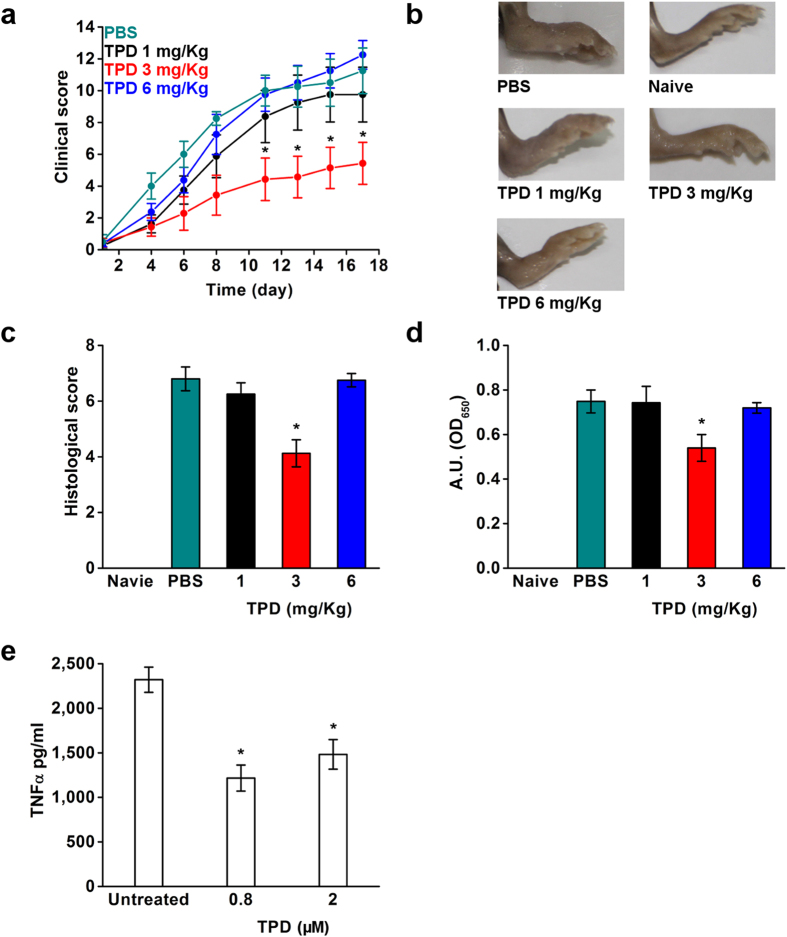
Treatment with TPD in collagen induced arthritis *in vivo* mice model and in human synovial cells. Collagen induced arthritis (CIA) in DBA/1 mice by immunization with type II collagen and intravenous injection of TPD 1 and 3 mg/Kg, 1 day after type II collagen boost and stopped after 10 days of daily injection. Macroscopic clinical score (**a**), representative photo of the inflamed joints (**d**), histopathologic analysis performed in hematoxylin/eosin-stained sections of joints (**c**) and serum from naïve, PBS control arthritic mice and TPD treated were analysed for anti-collagen antibodies (**d**). *P < 0.05, n = 10 (Mean ± SEM Student’s *t* test). (**e**) TPD incubation with synovial fluids derived from RA patient analyzed with human TNFα ELISA. *P < 0.05, n = 3 (Mean ± SD Student’s *t* test).

In the CIA model, only the mice group treated with 3 mg/Kg TPD displayed a significantly lower arthritis severity index (Fig. 3a), measured by the reduction in paw swelling compared to the PBS group (Fig. 3b). Correspondingly, the histopathological evaluation of the joints revealed protective effects in the 3 mg/Kg TPD group (Fig. 3c). The disease severity is correlated with the presence of serum antibodies specific to type II collagen, which is significantly reduced in the 3 mg/Kg TPD treatment group (Fig. 3d). Interestingly, no differences were found in the 1 and 6 mg/Kg TPD treatments, both groups show similar degrees of paw swelling compared to the PBS group (Fig. 3a,b). The macroscopic evaluation is in line with the histopathological score and the presence of type II collagen antibodies in these groups, showing comparable values as the PBS group (Fig. 3c,d). As TPD was shown to be effective in human cell systems, we evaluated its potential to modulate pro-TNFα maturation in human synovial fluid derived from RA patients. Cells were collected from human synovial fluid samples of a patient suffering from RA, and treated with LPS to induce secretion of TNFα. Cellular samples treated with TPD showed a significant reduction in TNFα levels in both concentrations 0.8 μM (1216.3 ± 146.6 pg/mL) and 2 μM (1482.4 ± 165.9 pg/mL) compared to the untreated controls (2320.3 ± 141.4 pg/mL) (Fig. 3e). Thus, these data demonstrate that modulating the maturation (and enzymatic activity) of membrane-bound TACE does affect control of TNFα release in a native system.

Overall, our results indicate that TPD treatment has a protective effect in animal models of IBD and RA. Interestingly, in both models we have found higher concentrations of TPD to be less effective in preventing disease progression, similarly to our observations with the LPS sepsis model described above. We do not yet know the reason for this bell-shaped pharmacological behavior, but suspect it may stem from dimerization or aggregation of TPD at high doses, or the induction of serum or tissue proteinases mediating its degradation *in vivo*, diminishing its disposition at the target site.

### Generation of monomeric, protease resistant TPD

**Figure 4:**
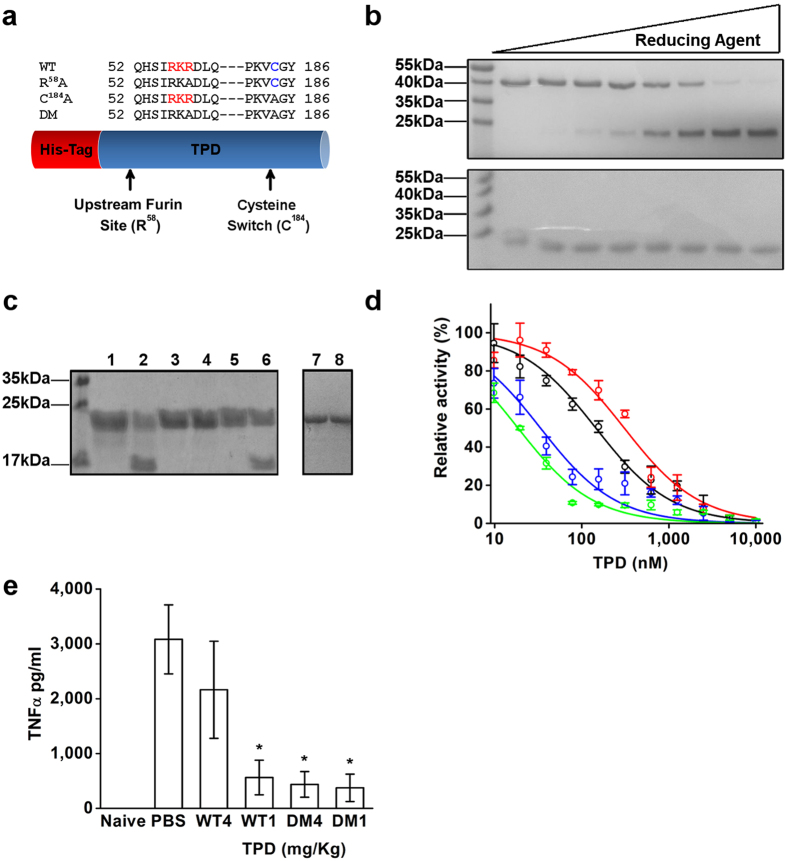
New mutants of TPD modulate TNFα secretion in LPS induced septic shock in mice. (**a**) Schematic representation of TPD, the red bar is the His tag and the blue bar is the TACE prodomain Asp^23^-Arg^214^. Marked in red is the furin cleavage site (RKR^58^) and in blue the cysteine residue in the cysteine switch (C^184^). The following mutants were created: R^58^A substituting residue 58 from arginine to alanine, C^184^A substituting cysteine 184 to alanine and a double mutant (DM) including both mutations R^58^A and C^184^A. (**b**) SDS PAGE analysis of the WT TPD (upper panel) and the C^184^A TPD (lower panel) with increase concentration of reducing agent. The WT TPD exhibits a molecular mass of 40 kDa corresponding to the covalent dimer which is switch to a 20 kDa band with the increase of reducing agent. In contrast, the C^184^A mutant display only a 20 kDa monomer independent to reducing agent concentration. (**c**) purified TPD WT and mutants incubated with furin protease and analyzed with SDS PAGE: TPD WT without furin (1) or with furin (2), TPD R^58^A without furin (3) or with furin (4), TPD C^184^A without furin (5) or with furin (6), TPD DM without furin (7) or with furin (8). (**d**) Inhibition curves of WT and mutants TPD on catalytic domain of TACE: WT (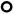), R^58^A (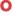), C^184^A (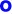) and DM (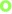). (**e**) Serum level of TNFα in naive C57/BL mice and C57/BL mice injected with LPS and treated with control PBS or different concentrations of TPD WT and DM (1, 4 mg/Kg). *P < 0.05, n = 6 (Mean ± SEM Student’s *t* test).

We set out to rationally design a monomeric and protease resistant form of TPD, using site directed mutagenesis to create a more stable protein and reduce its tendency for oligomerization. We first targeted the upstream furin cleavage site discovered in our lab (RKR^58^), with the specific substitution R^58^A, to increase resistance to proteolytic cleavage^21^. Next, we substituted the only cysteine residue in TPD, within the cysteine switch with the replacement C^184^A, to eliminate disulfide bond formation which may lead to formation of covalent dimers. Finally, the double mutant R^58^A/C^184^A was created, TPD-DM (Fig. 4a).

We examined the molecular properties of the TPD mutants by assessing their oligomerization and resistant to proteolytic degradation. The WT TPD migrates as a 40 kDa band in SDS-PAGE under non-reducing conditions, corresponding to the dimeric form. Increasing concentrations of reducing agent induce electrophoretic migration of TPD as a smaller band of around 20 kDa, corresponding to the monomeric form (Fig. 4b, upper panel). TPD C^184^A migrates exclusively as a monomer, independent of reducing agent concentration (Fig. 4b, lower panel). This result indicates that C^184^ mediates formation of disulfide-linked TPD dimers. The susceptibility of TPD mutants to proteolytic cleavage was tested by incubation with recombinant furin. Both WT and C^184^A TPD are cleaved by furin, whereas the R^58^A and DM mutants were resistant to that processing (Fig. 4c).

**Figure 5:**
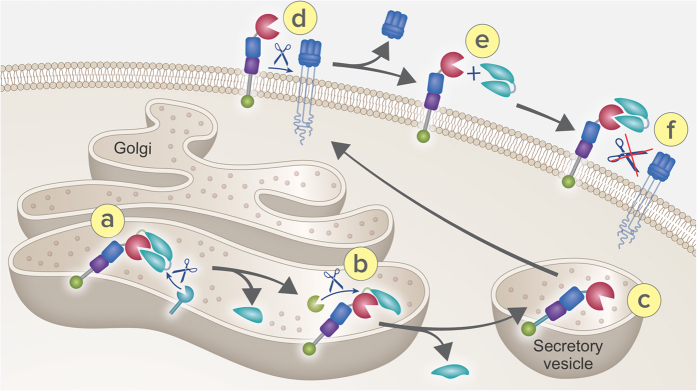
Regeneration of TACE zymogen using TPD. Schematic representation of TACE inhibition by TPD: TACE is generated as a zymogen form (**a**) which undergoes proteolytic cleavage of the auto-inhibitory prodomain in two furin sites by furin (**b**). The activated TACE is then delivered to the cell surface in secretory vesicle (**c**) to the cell membrane (**d**) to shedding its substrates such as TNFα. The addition of TPD restore the zymogenic form (**e,f**) inhibiting specifically its catalytic activity.

The inhibitory activity and selectivity of the TPD mutants were evaluated *in vitro*. The IC_50_ values of WT TPD and the R^58^A mutant were 145 ± 1.9 nM and 307 ± 33.6 nM respectively, indicating that removal of the furin cleavage site did not change drastically the inhibitory potency of the protein. Interestingly the C^184^A mutant and the double mutant resulted in an increase in potency, with IC_50_ values, 33 ± 4.6 nM and 18.5 ± 1.7 nM respectively (Fig. 4d). These results indicate that engineered, monomeric TPD bearing the C^184^A substitution is a more effective inhibitor of TACE activity. Moreover, the TPD mutants keep the selectivity towards TACE (Table 1). The TPD-DM mutant was functionally tested for modulation of TNFα levels by inhibiting TACE *in vivo* in the mouse septic shock model described above. WT or TPD-DM was injected at two doses, 1 and 4 mg/Kg. For the lower dose, both WT and TPD-DM showed a similar effect in lowering TNFα levels in serum (563 ± 315 pg/mL and 374 ± 249 pg/mL, respectively), a 6-fold reduction relative to PBS treated mice (3082 ± 629 pg/mL; Fig. 5e). A significant difference was observed for the higher dose where, WT TPD show no significant reduction in TNFα levels (2163 ± 885 pg/mL), but the TPD-DM mutant significantly reduces TNFα levels (437 ± 223 pg/mL) compare to the PBS control. Taken together, the TPD-DM (R^58^A/C^184^A) mutant showed higher inhibitory potency, resistance to furin proteolysis and prevention of oligomerization, resulting in increased efficacy in reducing TNFα levels *in vivo,* at higher doses, making it a superior inhibitory molecule.

## Discussion

Accumulating knowledge on the pathogenic mechanisms of various inflammatory or immune-mediated diseases has led to the development of anti-TNFα therapeutics. The latter represent treatment advances in a number of inflammatory conditions such as RA and IBD. Targeting cell surface and/or soluble TNFα *in vivo* thus offers a molecular strategy that contrasts with the pan immunosuppressive agents often used to treat various inflammatory diseases. Yet, such efficient interference with TNFα homeostasis is thought to contribute to the adverse effects resulting by TNFα inhibition *in vivo*^34^. Here we are proposing the following inhibitory mechanism by re-introducing TACE zymogen on the cell surface: TACE is translated as an inactive zymogen and likely delivered to the Golgi compartment in which the prodomain is being removed^21^ (Fig. 5). This process occurs in a sequential manner, where initial cleavage is performed at the furin upstream site (Fig. 5a), to generate an intermediate product which exposes the secondary boundary site, allowing removal of the residual prodomain (Fig. 5b). Activated TACE is then delivered to the cell surface, ready to shed its cognate substrates (Fig. 5c,d). By adding the TPD, the zymogen form is restored at the cell surface, preventing its shedding activity (Fig. 5e,f).

ADAM proteases are an important group of potential therapeutic targets^35^ and inhibiting the ADAMs depends on an accurate understanding of their exact function *in vivo*^36^. The unsuccessful clinical trials with inhibitors of MMPs suggest that specificity is a critical factor in drug development for these homologous class of enzymes^37^,^38^. The ADAMs are generated as zymogens with an auto-inhibitory N-terminus prodomain which evolved specifically with their catalytic counterpart. To date, there are only two high resolution structures available for MMP prodomain^39^,^40^, highlighting the intrinsically unstructured property of prodomains. This arrangement is likely to be even more complex for the ADAMs, having prodomains typically twice as long. The ADAM prodomains also participate in the folding and secretion of their associated catalytic doamains^41^,^42^,^43^. In addition, ADAM prodomains may have a complex fold including at least two independently folded sub-domains^24^,^44^. Utilizing the prodomains of these enzymes provides novel opportunities to rely on nature’s design to selectively inhibit members of these otherwise structurally homologous enzyme families.

Regenerating ADAMs isolated prodomains, and specifically TACE, is a challenging task due to the intrinsic properties of these domains resulting in an unstable refolded form^23^ or heterogeneous folded products^45^. In addition, while the refolded TACE prodomain is a nanomolar inhibitor for the catalytic domain, it is only a high micromolar inhibitor against the full ectodomain *in vitro*^23^ questioning its effectiveness. Here we successfully constructed a recombinant human TACE prodomain, which is natively folded, soluble and highly expressed in *E. Coli*. TPD contains substantial secondary structure with high β-strand content, in agreement with previous reports of ADAM prodomains^23^,^44^,^46^, and selectively inhibited the catalytic domain of TACE but neither the homologous MMP family members nor the closely related ADAM10. TPD also effectively reduced the maturation of pro-TNFα and hence decreased the secretion of TNFα in cells at micromolar levels, similar to TIMPs in cell based assays^47^,^48^. In contrast to TIMPs, which inhibit number of MMPs as well as ADAMs, TPD specifically arrested inducible TACE shedding activity, but not the constitutive shedding by ADAM10, its closest homologue.

The potential of using ADAM prodomain as a selective modulator has been demonstrated *in vitro* for TACE and ADAM10 by us and others in previous studies^23^,^46^. However, past studies did not proceed to examine the *in vivo* effect of the prodomain. Conceptually TPD represents a new biological tool for autoimmune diseases *in vivo* models. Overproduction of TNFα strongly correlates with diverse inflammation-related pathologies such as septic shock^49^, inflammatory bowel disease (IBD)^50^ and rheumatoid arthritis (RA)^51^. In this study, by using this restoration of TACE zymogen strategy, we showed that TPD is an efficient TNFα modulator as demonstrated in the successful treatment of the collagen-induced arthritis and TNBS-induced colitis models which both are dependent on TNFα as demonstrated in transgenic mice^52^,^53^. Thus, by directly preventing the shedding of TNFα by TACE, we have proved it to be feasible *in vivo*.

*In vivo* treatment with TPD was more efficacious using its protease resistant monomeric form. Mutants containing the cysteine to alanine mutation were less prone to aggregation, preventing the disulfide bonding which generates a covalently bonded dimer as seen in the WT. In addition, mutants with an abrogated furin site were resistant to furin cleavage *in vitro.* Mutants lacking an intact cysteine switch appeared to be more potent when examined as inhibitors for TACE, showing a decrease of 10 fold in IC_50_ values. Importantly, all variants maintained the specific inhibitor activity towards TACE. *In vivo* experiments using septic shock murine model demonstrated the favorable use of a mutated form of the TPD being effective in all concentrations administered. Most recently, the biological impact of this inhibitor was demonstrated on the modulation of CXCL12-CXCR4–induced motility and rapid stem and progenitor cell mobilization mediated *in vivo* by TACE^54^. Finally, treatment with TPD was shown to be also effective in attenuating TNFα levels in synovial fluid derived from RA patients.

Here we have demonstrated the application of exogenously added TPD to reconstitute TACE the zymogen form and prevent its shedding activity. The advantage of utilizing the inhibitory prodomain as an agent for specific targeting of ADAMs is evolutionarily logical considering that each prodomain evolves and is designed as an autoinhibitor/chaperone linked to its relative ADAM. Moreover, the prodomain inhibition mechanism combines chelating the active site zinc together with protein-protein^39^. The advantages of using prodomain are clear and may be extended to other ADAM prodomains e.g. ADAM10^46^, ADAM22^44^ that were stably expressed and isolated. Finally, our efforts were focused on the prodomain of TACE mainly because TACE was found to be a major sheddase of the pro-inflammatory cytokine TNFα that is mostly related to autoimmune disorders. However, following the successful data in the TNFα dependent autoimmune disease mice models, testing other TACE involved pathological states is a rational step. Seeing the strong association between TACE and shedding of the EGFR ligand family, it is only logical to consider cancer-related applications. Therefore, a cancer model, which is specifically effected by stimulated TACE shedding of EGFR ligand, would be suitable for testing TPD application.

## Methods

### Expression and purification of TPD WT and mutants

Electrocompetent *E. Coli* BL21 (DE3) were transformed with the corresponding TACE prodomain Asp^24^-Arg^214^ in pET28 plasmid with the DNA optimized sequence: ATGGACCCGGGCTTTGGCCCGCATCAGCGTCTGGAAAAACTGGATAGCCTGCTGTCTGATTATGATATTCTGAGCCTGTCTAACATTCAGCAGCATAGCGTGCGTAAACGTGATCTGCAGACCAGCACCCATGTGGAAACCCTGCTGACCTTTAGCGCGCTGAAACGTCATTTTAAACTGTATCTGACCAGCAGCACCGAACGTTTTAGCCAGAACTTTAAAGTGGTGGTGGTGGATGGCAAAAACGAAAGCGAATACACCGTGAAATGGCAGGATTTTTTTACCGGCCATGTGGTGGGCGAACCGGATAGCCGTGTGCTGGCCCATATTCGTGATGATGATGTGATTATCCGCATTAACACCGATGGCGCGGAATATAACATTGAACCGCTGTGGCGTTTTGTGAACGATACCAAAGATAAACGCATGCTGGTGTACAAAAGCGAAGATATCAAAAACGTGAGCCGTCTGCAGAGCCCGAAAGTGTGCGGCTATCTGAAAGTGGATAACGAAGAACTGCTGCCGAAAGGCCTGGTGGATCGTGAACCGCCGGAAGAACTGGTGCATCGTGTGAAACGT. The cells were grown at 37 °C, induced with 200 μM IPTG and grow at 15 °C overnight. Cells were harvested and resuspended with 50 mM Tris, 300 mM NaCl, 20 mM Immidazole, 0.1 mg/ml Lysozyme, 1 μg/ml DNA-ase and protease inhibitor cocktail (Roche), pH 8. The resusepended pellets were sonicated and centrifuged at 15000 rpm for 45 min at 4 °C. The supernatant was applied to a Ni^+2^ column (GE Healthcare) and eluted with buffer containing 50 mM Tris, 300 mM NaCl, and 250 mM imidazole, pH 8. The eluted protein was dialyzed against 50 mM Tris, pH 8 at 4 °C overnight and applied to HiTrap Q HP column (GE Healthcare). Elution was performed with salt gradient starting from 0 M NaCl 50 mM Tris, pH 8 to 1 M NaCl 50 mM Tris, pH 8 and fractions were collected and analyzed by 15% SDS PAGE. The fraction containing TPD was applied in included size exclusion chromatography using Superdex 75 (GE). The expression of the prodomain in this construct is in a soluble form, in contrast to previous reports^55^, where the expression was done in inclusion bodies followed by refolding step. The previous mutants of the refolded TPD could not exited 0.09–0.14 mg/ml concentrations after refolding, whereas the soluble prodomain could be concentrated to higher concentration for animal studies purposes.

### Circular Dichroism (CD)

CD measurements were performed using an Aviv 202 spectropolarimeter with a 1-mm path length quartz cuvette at 25 °C. Wavelength scans were done in the 190–260-nm far-ultraviolet range. The CD spectra of TPD were recorded at a concentration of 0.1 mg/ml in 150 mM NaCl, 50 mM Tris buffer, pH 8, at 2 s/nm. The data sets were averaged and then normalized to the baseline at 255 nm.

### Enzymatic assay and inhibition by TPD

The catalytic domain of TACE was produced in insect cells system as previously described^28^; the catalytic domains of MMP7, MMP9 and MT1-MMP are all expressed and purified in our laboratory and human ADAM10 was purchased from R&D. The enzymatic activities were tested by hydrolysis processing of fluoregenic peptide by purified TACE (10 nM) (Mca-PLAQAV-Dpa-RSSSR, 10 μM) (R&D system), MMP9 (10 nM), MMP7 (10 nM) and MT1-MMP (10 nM) (Mca-PLGL-Dpa-AR, 10 μM) (R&D system) and ADAM10 (Mca-KPLGL-Dpa-AR, 10 μM) (R&D system) at 37 °C monitoring the increasing fluorescence intensity at λ_ex_ = 340 nm and λ_em_ = 340 nm. The inhibition assay was carried out in a range of concentration of TPD (3 nM–2500 nM) after pre-incubation in 37 °C for 30 min. Initial reaction rates were measured and inhibition constant were evaluated by fitting to equation of IC_50_.

### Inhibition in cell based assays

Primary macrophages were extracted from the peritoneum after Thioglycollate injection in Balb/c mice. 50,000 cell/well in 96 wells were seeded in DMEM for overnight. The cells were incubated with 100 ng LPS and different concentrations of TPD: 0.25, 0.5 and 1 μM or 20 μM TAPI in DMEM media for 3 hours. TNFα concentrations in the media were measured with mouse TNFα ELISA kit (BD Bioscience) according to manufacture instructions. 1,000 CHO cells stably transfected with human TNFα and TACE were seeded overnight in 96 well and pre-incubated 2 hours with 1.25 and 5 μM of TPD and 20 μM TAPI (Enzo Life Sciences). The media was washed and replaced with fresh media containing the same substances. The conditioned media were collected after 4 hours and TNFα was measured with human TNFα ELISA kit (BD Bioscience) according to manufacture instructions.

### Neuropilin-1 differential cleavage in HEK293 and inhibition by TPD

HEK293 cells were grown in Nunc 6 well plate in DMEM media containing pen-strep and 10% FBS, 37 °C and 5% CO_2_ to ~80% confluence. Mouse full-length TACE WT and Neurophilin-1 were trasfected with jetPEI^®^ reagent (PolyPlus) according to manufacture instructions. After overnight incubation the media were replaced with a fresh media with or without TPD in different concentration. Cells were harvested and lysed after overnight in the conditioned media and analyzed by western blot. Equal protein concentrations were loaded in each sample of the in 8% SDS PAGE and transferred to a nitrocellulose membrane. The blots were probed with rabbit anti-Myc (for TACE) antibody or anti-HA (for Neurophilin-1). The Rabbit anti-Mouse conjugated horseradish peroxidase (Santa Cruz) was used as a secondary antibody. Signal was detected with ECL (Pierce).

### LPS-induced septic shock model

LPS inducing septic shock mouse model was done according to standard protocol^56^. Balb/c mice (age 7 week) were initially injected intravenously with PBS, 0.5, 1 and 2 mg/kg TPD one hour before peritoneum injection of 100 mg LPS (Sigma Aldrich) challenge. Each treatment group contained 8 mice. Blood samples were drawn 1.5 h following LPS-stimulation and TNFα levels in serum where determined by mouse TNFα ELISA kit (BD Bioscience) according to manufacture instructions. All animal studies were approved and carried out in accordance with the Weizmann Animal Care and Use Committee.

### Induction of TNBS colitis and treatment with TPD

TNBS (2,4,6-Trinitrobenzenesulfonic acid) (Sigma Aldrich) colitis was induced in female Balb/c mice ages 8–10 weeks as described^57^, control mice received 50% ethanol alone. TPD were injected intravenously daily for 7 days at 1 and 4 mg/kg, starting from day 0 (TNBS administration). As negative control mice were treated with vehicle PBS and positive control were treated with Dexamethasone. Each treatment group contained 10 mice. Macroscopic scoring of gross colonic damage, 7 days after TNBS administration was graded in a blinded fashion according to Reuter *et al*.^58^. Microscopic scoring: proximal, medial, and distal portions of colon were fixed in 10% phosphate-buffered formalin. Paraffin-embedded sections were stained with hematoxylin and eosin. The degree of histologic damage and inflammation was graded in a blinded fashion according to Elson *et al*.^59^. All animal studies were approved and carried out in accordance with the Weizmann Animal Care and Use Committee.

### Optical imaging of fresh tissues

TPD was conjugated to AnaTag Hilyte Fluor 750 (AnaSpec) according to manufacturer’s instruction. TNBS colitis induced mice were injected via tail vein with 1.5 nmol of TPD-HiLyte Fluor 750 (4.5 nmol equivalent Hilyte Fluor 750/mouse) or 4.5 nmol of Hilyte Fluor 750 only. One TNBS treated mouse was not injected and was used as a blank control. One group of mice sacrificed at 2 hours and the other group of mice was sacrificed at 10 hours after intravenous injection of fluorescent marker tagged TPD and fluorescent marker only. The dissected tissues (Heart, Lung, Kidney, Liver, Stomach, Spleen, Intestine, Colon) were imaged immediately. The mean fluorescent intensity of each tissue sample was obtained by subtracting the mean flourescence intensity of corresponding tissue from the blank mouse. The fluorescent intensities in the heart were used to reflect the fluorescent intensities in the blood. The colon to heart ratio of fluorescence was calculated. Fluorescence imaging was performed with an IVIS (IVIS^®^100/XFO-12, Xenogen Corp., Alameda, CA, USA). Near Infrared Fluorescence, in units of photons/sec, was detected using 710/50 nm and 800/75 nm filter sets for excitation and emission, respectively, with one-second-integration time.

### Collagen induced Arthritis treatment with TPD

DBA/1LacJ male mice were immunized on day 0 with 100 μg of bovine type II collagen (EPC) in CFA, and the mice were boosted with 100 μg of bovine type II collagen on day 21 according to previously described protocol^60^. Treatment with TPD 1, 3 and 6 mg/Kg or PBS was given intravenously beginning when 40% of animals developed signs of disease. Each treatment group contained 10 mice. Assessment of disease progression was done daily from the beginning of the treatment in blind fashion according to establish scoring system described by Brand *et al*.^60^. Mice were sacrificed at day 18 and the back limbs were decalcified fixed in 10% phosphate-buffered formalin. Paraffin embedded sections were stained with hematoxylin and eosin. The degree of histological damage and inflammation was graded in a blinded fashion according to Williams^57^,^61^. All animal studies were approved and carried out in accordance with the Weizmann Animal Care and Use Committee.

### Measurement of Anti-collagen IgG

Blood was taken from the PBS and treated collagen induced arthritis mice model for testing the titter for anti-collagen antibody. 96 wells plate (Nunc) was coated with type II collagen (EPC) at 5 μg/mL. The plate was blocked for 1 h at room temperature with 2% BSA. The sample serums were incubated (diluted in PBS/Tween-20) for 2 hour at room temperature including a standard serum sample. Levels of anti-collagen IgG may vary enormously between mice and it is important to serially dilute samples to ensure that comparisons are made based on the linear portion of the titration curve. Detection of the bound anti-collagen antibody to the type II collagen coating was done with rabbit anti-mouse conjugated horseradish peroxidase using TMB (Thermo Scientific) as substrate. The plates were read with ELISA standard plate reader at 630 nm.

### Secretion of TNFα in synovial fluids in RA patient and inhibition by TPD

Synovial fluid was obtained from RA patients. Cells were pellet by centrifugation 800 × g (2200 rpm), 15 min, 25 °C, washed in PBS and counted. Cells were seeded in 48 well plate (750,000/well, in 500 ul DMEM + 10%FCS + PSG) and incubated 72 hours at 37 °C, 5% CO_2_, with 0.8 and 2 μM TPD and media were collected and analyzed with human TNFα ELISA kit (BD Bioscience) according to manufacture instructions. All experiments were performed in accordance with guidelines and regulations of Sourasky Medical Center and the Sackler Faculty of Medicine.

### Furin cleavage of TPD mutants

Purified TPD WT, R58A, C184A and DM mutants (0.2 mg/ml final concentration) were incubated with furin (2 Enzyme Unit New England Biolab) in furin assay buffer (100 mM HEPES (pH 7.5), 0.5% Triton X-100, 1 mM CaCl_2_, 1 mM 2-mercaptoethanol) in 37 °C for three hours. 10 μl of each reaction was analyzed using 12% SDS PAGE.

## Additional Information

**How to cite this article:** Wong, E. *et al*. Harnessing the natural inhibitory domain to control TNFα Converting Enzyme (TACE) activity *in vivo. Sci. Rep.* **6**, 35598; doi: 10.1038/srep35598 (2016).

**Publisher's note:** Springer Nature remains neutral with regard to jurisdictional claims in published maps and institutional affiliations.

## Figures and Tables

**Table 1:** Summary of IC_50_ of TPD WT and mutants in different MMP enzymes (μM, means ± SD).

Mutant	TACE	MT1	MMP7	MMP9
WT	0.145 ± 0.011	ND	ND	ND
R^58^A	0.307 ± 0.033	4.653 ± 1.481	ND	4.337 ± 2.747
C^184^A	0.033 ± 0.004	ND	ND	ND
DM	0.018 ± 0.001	ND	5.443 ± 0.865	ND
